# Mycobacterial Genetic Technologies for Probing the Host-Pathogen Microenvironment

**DOI:** 10.1128/iai.00430-22

**Published:** 2023-05-30

**Authors:** Oyindamola O. Adefisayo, Erin R. Curtis, Clare M. Smith

**Affiliations:** a Molecular Genetics and Microbiology, Duke University, Durham, North Carolina, USA; University of California at Santa Cruz

**Keywords:** tuberculosis, mycobacterial genetics, host-pathogen interactions, infection microenvironment

## Abstract

Mycobacterium tuberculosis (*Mtb*), the causative agent of tuberculosis, is one of the oldest and most successful pathogens in the world. Diverse selective pressures encountered within host cells have directed the evolution of unique phenotypic traits, resulting in the remarkable evolutionary success of this largely obligate pathogen. Despite centuries of study, the genetic repertoire utilized by *Mtb* to drive virulence and host immune evasion remains to be fully understood. Various genetic approaches have been and continue to be developed to tackle the challenges of functional gene annotation and validation in an intractable organism such as *Mtb*. *In vitro* and *ex vivo* systems remain the primary approaches to generate and confirm hypotheses that drive a general understanding of mycobacteria biology. However, it remains of great importance to characterize genetic requirements for successful infection within a host system as *in vitro* and *ex vivo* studies fail to fully replicate the complex microenvironment experienced by *Mtb.* In this review, we evaluate the employment of the mycobacterial genetic toolkit to probe the host-pathogen interface by surveying the current state of mycobacterial genetic studies within host systems, with a major focus on the murine model. Specifically, we discuss the different ways that these tools have been utilized to examine various aspects of infection, including bacterial survival/virulence, bacterial evasion of host immunity, and development of novel antibacterial/vaccine strategies.

## INTRODUCTION

Mycobacterium tuberculosis (*Mtb*) is an obligate intracellular pathogen that has adapted to life within a host. Within the host cellular niche, *Mtb* has evolved multiple mechanisms that drive bacterial survival, virulence, and immune evasion (1–3). The balance between *Mtb* and complex host microenvironments ultimately drives the variability of tuberculosis disease pathology and outcome (4). To understand the mechanisms that underlie pathogenesis, it is necessary to be able to directly probe bacterial responses within these diverse host environments. However, analyzing bacterial mechanisms driving infection within the various *Mtb* disease model systems remains technically challenging and largely under studied. In this review, we (i) highlight recent approaches within the mycobacterial “genetic toolkit” that have been utilized in elucidating host-pathogen interactions and (ii) discuss new approaches to bacterial gene virulence discovery and gene validation within host organisms.

## THE MYCOBACTERIAL GENETIC TOOLKIT: A BRIEF HISTORICAL BACKGROUND OF GENE-EDITING TECHNOLOGIES IN MYCOBACTERIA

Gene-editing and transcription modifying strategies, including DNA transformation, phage transduction, transposon insertion, recombineering, and more recently, CRISPR-based technologies (5–9) that generate gene knockouts, introduce point mutations, tag genes, or regulate gene expression within the mycobacterial genome have been instrumental in efforts to identify the bacterial genes that drive infection and are required for virulence. Despite the success of these techniques, the slow growth and low transformation efficiency of *Mtb* directs the continual effort to improve the ease and efficiency of generating genetic modifications. Moreover, these characteristics of *Mtb* contribute to the technical difficulty of simultaneously targeting multiple genes, which has ultimately hampered efforts to examine gene networks, redundant genes, and multigene interactions. Efforts to address this limitation have been recently developed and described and are largely based on CRISPR systems. These include single-step generation of multigene knockouts (10), as well as multigene repression systems (11–15). Altogether, mycobacterial genetic tools have been widely applied in *in vitro*, *ex vivo*, and *in vivo* systems and have greatly contributed to insights of mycobacterial physiology, virulence, and host response.

While targeting single genes has aided in functionally defining the mycobacterial drivers of infection and disease, this remains a low-throughput mechanism. As such, unbiased genome-wide approaches have proven useful in characterizing gene functionality and essentiality. Methods that have been at the forefront of genome-wide gene essentiality analysis of *Mtb* are Transposon Insertion Mutagenesis (TIM) techniques, such as Transposon Sequencing (TnSeq), which involve generating a bacterial library through phage-mediated delivery of transposon elements that integrate at TA dinucleotide sequences and subsequent sequencing to confirm transposon disruption (7, 16). Multiple TIM studies have aimed to define the regions of the mycobacterial genome conditionally essential under various *in vitro*, *ex vivo*, and *in vivo* conditions (16–20). However, as transposon insertion disrupts gene structure leading to loss-of-function mutants, TIM studies cannot probe genes that are indispensable for growth and reproduction, as these mutants do not survive the initial creation of the library. To circumvent this limitation, multiple tools have been developed that can target both essential and nonessential genes by conditionally regulating either gene or protein expression. One subset of tools leverages regulated gene expression through inducible promoters (21–26) and have been largely utilized in target-based whole-cell screening (TB-WCS) to screen drug compounds (27). Alternatively, the development of hypomorphs/degron mutants allows control of protein levels through conditional proteolysis of the target (28, 29). Furthermore, repressible gene expression has been combined with regulated proteolysis to precisely control protein expression (30). The recently developed CRISPR interference (CRISPRi) platform has also expanded abilities to target essential genes individually or as a library by transcriptionally silencing gene expression using the targeting specificity of the CRISPR-Cas system (11, 13, 31). Like the single gene targeting techniques, these approaches have been leveraged to analyze bacterial gene essentiality under various *in vitro* conditions.

The utility of the mycobacterial gene toolkit is readily apparent and explored in bacterial culture, but its application in surveying the host-bacterial interface at the site of infection is yet to be fully realized. As such, we aim to highlight efforts in the field to leverage bacterial genetic techniques that identify and characterize the mycobacterial genetic drivers of disease within complex host systems.

## MODELS OF TB DISEASE

As an obligate pathogen uniquely adapted to survival within host cells, studying the bacterial response within the host system remains an important aspect of understanding *Mtb* biology. Multiple models have been developed for studying *Mtb* within host cells from mammalian cell culture models to whole-animal models.

### Mammalian cell culture models.

As macrophages serve as the primary residence of *Mtb* bacilli, there is a great interest in studying the role of this cell type during infection. As such, tractable *ex vivo* macrophage cell culture models have been invaluable in understanding the genetic repertoire *Mtb* utilizes to survive within this highly relevant host cell type. These studies often leverage primary macrophages derived from human monocytes or murine bone marrow, as well as numerous human and murine immortalized cell lines such as THP-1 or RAW264.7 (32–34). However, evidence shows that (i) *Mtb* can infect multiple cell types during infection, including neutrophils, dendritic cells, and nonimmune cells (35–37); and (ii) macrophage immune polarization, cellular lineage, and metabolic state can drive differential responses to *Mtb* (38, 39). Currently, not all cell types are amenable to growth in cell culture or are difficult to obtain in large enough quantitates, complicating their analyses *ex vivo*. Various cell culture techniques that expand the complexity of cell-based models, such as tissue explants (40–42), provide insights into mammalian cellular interactions that underlie *Mtb* infection. However, these models do not lend themselves to long-term infections precluding the analysis of certain aspects of Mtb infection. Nevertheless, cell culture models have been successfully employed in identifying bacterial virulence determinants (43–45) and have been proven to be highly valuable tools with results that translate to a better understanding of host infection.

### Nonmammalian animal models.

Numerous nonmammalian models of infection have been developed and extensively utilized to study dynamics of various infectious agents (46). In the case of mycobacterial infection, these animal models include Caenorhabditis elegans as a model for Mycobacterium avium (47) and both leopard frogs and Drosophila melanogaster as models for Mycobacterium marinum (48, 49). However, the natural infection of zebrafish with M. marinum remains the most broadly utilized nonmammalian model of mycobacterial infection. This model has been successfully used to discover both host and mycobacterial factors driving disease pathogenesis that have been validated in other models (50, 51). In addition, the zebrafish model has played an important role in modeling certain important aspects of mycobacterial infection, including the dynamics of granuloma formation (52–54) and bacterial persistence (55, 56).

### Mammalian animal models.

Various mammalian models are currently used to investigate host-bacterial pathogenesis. Nonhuman primates (NHP), such as macaques (57, 58) and marmosets (59), are the closest model to human infection. However, NHP studies remain expensive and technically challenging, and as such, smaller mammalian models remain necessary to investigate narrow aspects of host response during infection. Rabbits and guinea pigs have long been used to study *Mtb*, but there remains a dearth of tools to examine the host immunological and genetic contributions to infection. Nevertheless, as both rabbits and guinea pigs develop disease pathology comparable to human infections, including the formation of caseous granulomas and, in the case of rabbits, similar pharmacokinetics to humans and the development of bacterial latency, they have proven to be attractive models for drug testing studies (60–63). While macaques remain the animal model of choice to explore coinfections of *Mtb* and HIV/AIDS (64, 65), guinea pigs have proven to be useful for examining *Mtb* comorbidity with type 2 diabetes (T2D) (66). Other mammals that have been tested as models for *Mtb* infection but have yet to be explored extensively include various rat species (67, 68), ferrets (69), mini pigs (70, 71), and the Chinese tree shrew (72).

The most extensively used *Mtb* animal model has, however, been the mouse. Classic inbred murine models have the benefit of being relatively cost-efficient and genetically tractable, with a ready availability of immunological reagents, close homology to many human genes, and study reproducibility. However, there are major differences in disease progression between murine and human TB disease that limit the utility of using the model in studying specific aspects of human disease. Unlike in human TB, the widely used inbred C57BL/6 (B6) and BALB/c mice (i) canonically lack caseous, necrotic granulomas that are a staple of human disease and (ii) support a chronic, persistently high number of bacilli in their lungs. To address these limitations, various mouse populations, including congenic and knockout models, have been developed. The C3HeB/FeJ (C3H) mouse strain has been shown to develop distinctive lesions that range from caseous necrotic lesions to the more commonly observed cellular inflammatory lesions (73) and as a result has been shown to respond differentially to drug regimens that are directly attributable to the formation of necrotic granulomas (74, 75). However, as C3H mice are highly susceptible to *Mtb* infection, the B6J.C3H-sst1 mouse, which carries the C3H *sst1* locus that has a dominant role in the development of necrotic lesions (76), has been developed and proposed as an alternative granuloma-forming mouse model. Specific targeting of the *Sp140* gene within the *sst1* locus phenocopies the susceptibility of the B6J.C3H-sst1 mouse, yielding another murine model of necrotic lesions (77). Another mouse model developed to mimic the human immunopathology of TB disease more closely is the humanized mouse model (HuMc), which includes NOD-SCID/γ_c_ null mice engrafted with human fetal liver and thymus tissue. These mice have been shown to develop necrotic lesions that are uniformly distributed among the population of mice (78) and has been shown to replicate results observed in clinical studies on the effectiveness of different drug regimen (79). HuMc have also shown great utility in the study of *Mtb* and HIV coinfections (80, 81). The HuMc model is, however, limited by cost, the percentage of reconstitution, and the presence of residual cells of murine origin, which may interfere with the immune response from the reconstituted human cells (79).

To better mimic and explore the role of host genetic diversity in disease, the Collaborative Cross (CC) recombinant inbred panel was developed, expanding the genetic diversity of mice while maintaining the reproducibility of inbred mouse strains (82). Since its development, the CC panel has been shown to exhibit a broad range of responses to *Mtb* infection (83) and can be leveraged to explore both host and bacterial determinants of infection. Another genetically diverse mouse cohort maintained as an outbred population is the Diversity Outbred (DO) panel, which reflects the heterozygosity seen in human populations (84, 85). Similarly to CC mice, *Mtb* infection of the DO panel is characterized by a diversity of response (86), and both panels have been used to assess protective responses to BCG vaccination (87, 88).

For a more comprehensive review of the animal models of TB infection, refer to Bucsan et al. (89) and Yang et al. (90). While these different models have their advantages and disadvantages and serve specialized roles in the analysis of *Mtb* infection, mice remain a favored model for examining complex host environments based on cost-effectiveness, tractability, and the comparative ease of utilization.

## MYCOBACTERIAL PATHOGENIC GENE DISCOVERY WITHIN THE HOST

Multiple *in vitro* and *ex vivo* experiments have been designed to exert unique selection pressures that mimic specific conditions within the host (e.g., standard conditions versus hypoxic conditions). However, these systems lack the ability to fully replicate conditions within the host over the length of a long-term infection. It is also evident that each unique host system even within the murine model (e.g., B6 versus BALB/c mice) exerts different pressures as evidenced by the difference in disease traits (83, 89–91). The subtle differences in the host environment exert unique pressures on *Mtb*, changing the genetic requirements for survival. This raises the question of the experimental approaches that can be taken to define the mycobacterial effectors of disease that can be targeted to improve antibacterial strategies while accounting for host diversity. Analyzing the biology of *Mtb* infection from its genome has played a key role in the identification of the bacterial genes required for, among other things, survival, virulence, and immune evasion. The following sections address unbiased genome-wide approaches.

### Bacterial survival and virulence.

To facilitate the simultaneous analysis of *Mtb* mutants, signature-tagged mutagenesis (STM) was adapted for use in *Mtb*. STM libraries are low-complexity libraries generated using phage containing a unique DNA tag that is incorporated into the bacterial genome and can be analyzed for abundance under different conditions (92, 93). In *Mtb*, STM has been used to identify virulence genes, including those involved in the synthesis of the cell wall lipid pthiocerol dimycocerosate (PDIM) (94), macrophage entry, and resistance to phagosome-lysosome fusion (95). STM studies also identified genes required to counter immune responses to interferon-γ (IFN-γ) but not inducible nitric-oxide synthase (iNOS) (96). The STM screen conducted by Cox et al. (94) and Camacho et al. (97) served as one of the first studies subjecting an *Mtb* transposon (Tn) library to murine selection. The identification of PDIM as a major virulence factor within the host by Cox et al. (94) was based on a library composed of 576 mutants of the Erdman lineage injected intravenously into B6 mice. Camacho et al. (97) similarly identified lipid biosynthesis genes from a screen of 1,927 MT103 mutants injected intravenously, within a 50-kb region starting with *fadD26* as playing a role during host infection. Using improved approaches based on microarray technology, Sassetti and Rubin (98) conducted transposon site hybridization (TraSH) analysis of a fully saturated H37Rv mutant library composed of 100,000 Tn mutants to identify 194 genes required for growth in B6 mice. TraSH screening allowed significantly larger and more complex pools of Tn mutants to be screened while lessening the labor required to conduct such screens. The authors highlighted the existence of kinetically distinct phenotypes among the family of *mce* genes. These results revealed the development of specialized function in gene families that had evolved through gene duplication as opposed to horizontal gene transfer. In a follow-up to Sassetti’s findings, Joshi et al. (99) investigated gene networks involving *mce* loci by utilizing a genetic interactions approach. To do this, the authors created a Tn library in Δ*mce* strains and identified genes necessary for survival in the absence of *mce* in B6 mice. Findings provided valuable insight into the *mce* gene networks and further confirmed kinetically distinct functions of duplicated *mce* loci. Nambi et al. (18) used a similar genetic interaction approach by leveraging the power of TnSeq to define the *Mtb* network responsible for defense against host oxidative pressures encountered during infection. In a study that compared gene essentiality in B6 mice, two distinct TnSeq libraries were created in wild type and *ctpC*::*hyg*, a superoxide-sensitive strain, which identified the ability of the superoxide dismutase SodA to form stable complexes with previously uncharacterized DoxX and SseA to recycle thiol radicals. In an expansive study, Smith et al. (83) defined the mycobacterial genetic repertoire required for survival and virulence of *Mtb* across a variety of genetically diverse hosts by combining a comprehensive library of *Mtb* Tn mutants with host variation of the Collaborative Cross. In this study, they found that while the number of *Mtb* genes required in each mouse genotype was largely similar, the composition of the gene sets varied widely across the 65 strains of mice. This indicated that the bacterial genetic requirement for survival was affected by the host genotype. As such, the number of genes required for bacterial survival was found to be expanded from about 214 in B6 mice to approximately 750 bacterial genes required for growth across the diverse host panel, highlighting the importance of host genetic variability when assessing the host-pathogen microenvironment. To maintain the complexity of the Tn library, most TnSeq studies utilize the intravenous route; however, Payros et al. (100) defined the role of Rv0180c in establishing infection early within the lung with a high-dose intranasal infection.

For genes that are essential for *Mtb* survival and thus cannot be targeted for knockout, it remains difficult to deconvolute their role in general bacterial survival and virulence in the host. Exploring the deployment of a tetracycline (Tet) regulated CRISPRi gene repression system to drive unbiased whole-genome screening in host cells was explored by Babunovic et al. (45) in murine bone marrow-derived macrophages (BMDMs). In this study, the authors validated mycobacterial genes previously shown to be essential for intramacrophage growth. In addition, the study revealed novel reduced requirements for the essential quinone synthase *menH* in macrophages compared to axenic culture growth, as well as defined an increased requirement for bacterial cholesterol import and metabolism genes when *Mtb*-infected macrophages were treated with all-*trans*-retinoic acid (ATRA). By harnessing the native *Mtb* CRISPR-Cas10 system, Rahman et al. (13) performed a genome-wide RNA interference (RNAi) screen of mycobacteria in THP-1 cells and identified 29 genes as required during macrophage infection, with many of those genes being in concordance with previously published reports. However, as this system is unregulated, its utility in targeting essential genes is yet to be confirmed. Consolidating the results of these CRISPRi studies provides an experimental basis for the utilization of genome-wide RNAi in animal models with the goal of examining drivers of bacterial virulence *in vivo*.

### Bacterial evasion of host immunity.

**FIG 1:**
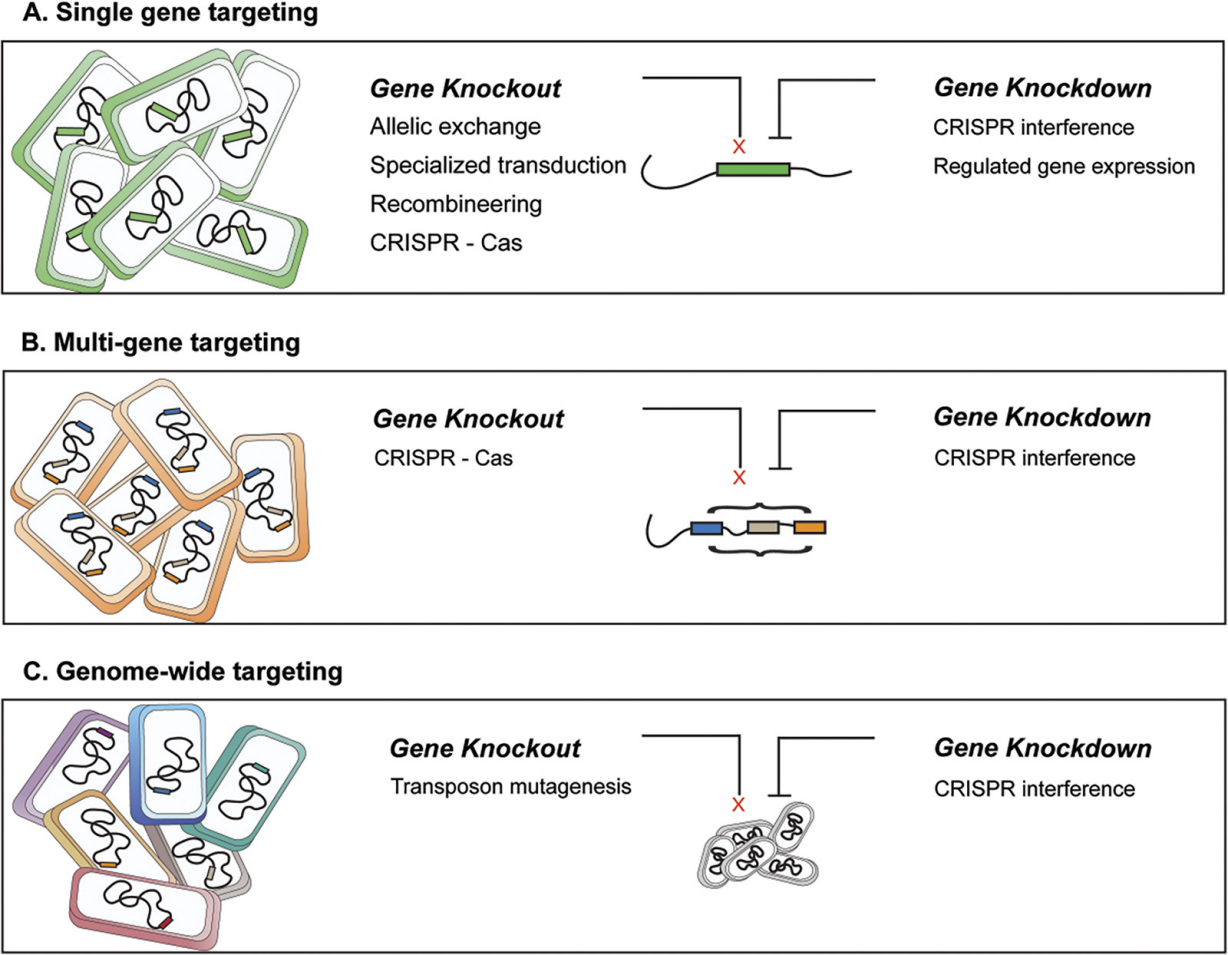
Gene editing techniques currently utilized in the genetic manipulation of *Mtb*. Researchers use various techniques to assess the contribution of specific *Mtb* genes to virulence and survival. Highlighted here are the broad categories of technologies contained within the mycobacterial toolbox used to individually target single genes, simultaneously target multiple genes or collectively assess genetic mutant pools targeted on a genome-wide scale. (A) The majority of the technologies available for targeting *Mtb* genes enable the creation of single genetic mutants (gene knockout) or the modulation of single gene expression (gene knockdown) by both repression and regulated induction. Due to the intractable nature of *Mtb*, efforts continue in technology development within the listed categories. These include endeavors to broaden recombineering substrate leading to the development of tools such as oligonucleotide-mediated recombineering followed by Bxb1 integrase targeting (ORBIT), as well as the assessment of the utility of various CRISPR-Cas systems for use in *Mtb*. (B) While many of the tools developed for single gene targeting can be harnessed to sequentially target multiple genes, these processes remain laborious and time-consuming. Fewer tools have been developed that can be used to simultaneously target multiple genes, and the available ones are based on various CRISPR-Cas systems for gene knockout or CRISPR interference for gene knockdown. (C) Unbiased genome-wide approaches that allow the analysis of many mutants within a single experiment are valuable but currently limited in developed technologies. Transposon mutagenesis has seen the most use, particularly within animal models, but CRISPR interference systems are being developed that have and will continue to further expand mycobacterial genetic analyses.

Beyond screening the genes in the *Mtb* genome for essentiality and virulence during infection, it is crucial to understand the bacterial genetic drivers of host immune evasion and disease pathology. Numerous mechanisms by which mycobacteria evade and manipulate the host immune response have been extensively studied and reviewed (Fig. 1) (3, 101–103). Nonetheless, there remains a gap in our understanding of the interaction of the bacteria with various host cell populations *in vivo* and the bacterial genes that mediate these interactions. Most of the studies that have defined these mycobacterial mechanisms have been conducted in *ex-vivo* systems, which has been recently reviewed by Ankley et al. (3). Leveraging global genetic approaches to address this question *in vivo*, studies have shown that by comparing *Mtb* Tn library profiles between host immunological KO models and classic murine models, researchers can investigate how specific host genes exert selective immune pressure on the bacteria. Murry et al. (104) performed a TraSH screen that utilized KO mice unable to produce either iNOS or IFN-γ, both of which play important roles in host response to *Mtb* (105). Mutations that interrupted transport of cell wall lipid PDIM were more attenuated in iNO_S_^−/−^ B6 mice compared to B6 mice. Since nitric oxide (NO) is known to suppress IFN-γ production, it was hypothesized that the PDIM transport mutant was hypersusceptible to elevated IFN-γ levels in iNOS^−/−^ mice. However, as the mutant did not gain a growth advantage compared to wild-type (WT) bacteria in IFN-γ*^−/−^* mice, it indicated that PDIM transport mutants are sensitive to host responses independently of IFN-γ. With the advent of next-generation sequencing (NGS) and the development of TnSeq, Zhang et al. (106) utilized a fully saturated TnSeq library in major histocompatibility complex (MHC) knockout B6 mice to study the effect of CD4^+^ T cells on M. tuberculosis. They identified a mechanism in which *Mtb* can utilize *de novo* tryptophan synthesis in response to the efforts of the host CD4^+^ T-cell attempts to starve the bacteria of tryptophan. Beyond defining the mycobacterial genetic fitness requirements across diverse hosts, Smith et al. (83) also quantified genome-wide association of host immunological phenotypes and bacterial fitness traits to explore the complexity of host immunological state on bacterial genetic requirement. This study identified bacterial gene modules associated with particular host immune responses. The specific host pathways underlying these bacterial traits is an area for future follow-up. Altogether, these studies establish a further basis for whole-genome screens to investigate both the bacterial determinants of survival/virulence, as well as immune evasion, particularly through the distinct stages of infection.

### Antibacterial therapies.

A fundamental goal of the TB field today is the improvement of current TB therapies, which include the development of antibiotics with new targets and modes of action (MOAs), the establishment of effective drug regimes, and finding new or improving vaccine strategies. The major approaches to drug discovery are: (i) screening drugs for whole-cell activity against the bacteria, (ii) identifying promising bacterial targets to design drugs against, or (iii) some combination of both approaches. Effective drug targets discovered *in vitro* do not necessarily translate to effective drug targets for *in vivo* use (107–109). However, it remains technically difficult to screen large libraries of drugs against *Mtb in vivo*. To combat this disconnect, several studies have screened for novel antibiotic drug targets by introducing Tn libraries to drug-treated murine models. One such study by Bellerose et al. (110) performed TnSeq in BALB/c mice treated with the standard HRZE antibiotic regimen of isoniazid (INH), rifampin (RIF), pyrazinamide (PZA), and ethambutol (ETH) to elucidate factors that limit antibiotic efficacy in *Mtb* treatment. This resulted in identification of *glpK* as a controller of both glycerol metabolism and antibiotic efficacy. In a follow-up study, Bellerose et al. (108) additionally investigated the unique mechanisms that alter drug efficacy in each of the individual antibiotics that compose the standard HRZE regiment. TnSeq was completed in infected BALB/c mice treated with INH, RIF, PZA, or EMB and analyzed at different time points post-treatment. Unique genes were identified that affected the cost of fitness for each treatment, with most mutations altering susceptibility to only one antibiotic. Notably, there was a lack of similarity between mutations predicted to alter antibiotic susceptibility *in vitro* versus *in vivo.* These studies support the feasibility of combining genome-wide targeting techniques with antibiotic treatment to identify additional genes or pathways that could be targeted to potentiate or complement drug activity *in vivo*. While there remains a gap in identifying drug and vaccine targets within model host systems, a recent study that utilized an unbiased whole-genome CRISPRi approach to screen for bacterial genes whose incomplete inhibition resulted in significant fitness costs and would thus be more vulnerable to drug targeting (111) presents potentially innovative paradigms for assessing appropriate gene targets *in vivo*.

## ANALYZING AVAILABLE DATA SETS

Many of the mycobacterial gene discovery studies generate large amounts of data that could serve as valuable resources for downstream identification and analyses of gene targets with important roles during infection. However, most of the information remains difficult to effectively query and compare across studies, and as such, various resources and databases are being developed to increase access to the data sets. This includes the development of a database that combines publicly available data from TnSeq screen in a central repository called *Mtb* transposon sequencing database (MtbTnDB; https://www.mtbtndb.app/) (112) and the development of a tool that aids in the design, analysis, and sharing of CRISPRi experiments developed by the Rock lab (https://pebble.rockefeller.edu). Altogether, as this compendium of data is collated and deposited, new insight will be gained through the capacity to directly compare *Mtb* genes across experimental conditions, cell types, host models, bacterial strains, and time points.

## MYCOBACTERIAL GENE VALIDATION WITHIN THE HOST

Using unbiased genome-wide genetic approaches to drive gene discovery is the first step in defining the bacterial effectors of pathogenesis. Validating the pathogenic role of identified genes is the next essential step to inform *Mtb* elimination strategies. Characterization of *Mtb* gene function within the host is of particular importance as genes may have multiple roles dependent on environmental factors, infection route, and disease kinetics. Most validation studies utilize single gene knockout strains that provide an easily interpretable result that can be easily validated by genetic complementation. However, considering the slow growth of M. tuberculosis, the relative difficulty of generating genetic mutants and the length, labor, and expenses involved in whole-animal experiments, there is a great interest in testing multiple mutants in a single aerosol experiment, which will be subsequently discussed.

### Validating: bacterial survival and virulence.

The analysis of single knockout *Mtb* mutants in animal models has provided a depth of knowledge on the survival and virulence roles of numerous *Mtb* genes during infection. An excellent review from Ehrt et al. (2) summarizes some of these studies. There is, however, a steady increase in the analysis of genes through regulated gene expression to study bacterial survival and virulence during both acute and chronic phases of infection as this system provides the advantage of being able to target essential genes. One of the first studies to verify the utility of Tet-regulated systems in *Mtb* during infection was done by Gandotra et al. (113). The authors sought to validate the *in vitro* essentiality of the proteasome genes *prcA* and *prcB* initially identified in a TnSeq study (98) by regulating the gene expression of both genes in both *in vitro* and *in vivo* conditions using both tetracycline-inducible (TET-ON) and tetracycline repressed (TET-OFF) promoters. The authors found that the conditional loss of *prcA* and *prcB* expression was essential for the full virulence of *Mtb* in both axenic and murine growth. Interestingly, the requirement for the genes was mainly observed during the chronic phase of mouse infection, suggesting the conditional essentiality of these genes in response to the host environment. Another study that utilized regulated gene expression to analyze the role of an essential gene in bacterial survival or virulence during host infection was conducted by Stallings et al. (114); the essential gene *carD* was expressed under a TET-OFF promoter and revealed the relevance of the gene in establishing and maintaining infection in B6 mice. Repression of *carD* expression either during the acute phase of infection or after the establishment of infection led to death of *Mtb* in both cases. While most regulated gene expression studies in mice utilize the small-molecule tetracycline inducer, doxycycline, the Nandicoori lab showed the essential role of both kinases in establishing an infection in B6 mice in back-to-back studies (115, 116) by placing the essential genes *pknA* and *pknB* under a pristinamycin-inducible promoter (pptr-ON). Using a dual system of conditional gene regulation that combined inducible gene expression with regulated proteolysis, Kim et al. (30) showed that loss of the essential gene *nadE* at different points during mouse infections led to bacterial clearance and a reduction in lung pathology, which indicated a potential role for this enzyme during persistence and as a potential drug target. Moreover, Su et al. (117) utilized the regulated dual control system to study mycobacterial persistence in B6 mice. In this study, the authors transiently depleted the essential genes *birA* and *trxB2* and observed the development of latency and bacterial growth relapse in a gene-dependent manner, which indicated that targeting essential genes might not always lead to bacterial clearance. To enhance the utility of regulated gene expression, CRISPR-based systems have been investigated to simultaneously regulate the expression of multiple mycobacterial genes within a single bacterium. A recent study reports the use of Cas9-based CRISPRi in mycobacteria to investigate the transcriptional repression of the candidate genes *mmpl3* and *qcrB* within the THP-1 cell line (118). Using a Cas12a-based CRISPRi system, Fleck and Grundner (11) showed the simultaneous repression of luciferase genes encoded in the mycobacterial genome and the consequent loss of luminescence in THP-1 cells. These studies indicate the potential of regulated gene expression systems to drive gene validation studies to examine the roles of gene networks or essential genes in bacterial survival and virulence during infection within model host systems. However, the conditional regulation of genes come with the requirement of maintaining the optimal level of inducer across animal tissue and the possibility of loss of control and regulation (114, 119) especially over the length of a murine *Mtb* infection that can last many months. Further, while the discussed CRISPRi studies in THP-1 cells indicate the absence of an effect on the health of the cell line, their utilization for long-term studies in mice might be hampered by shortcomings, including detrimental health effects of the small-molecule tetracycline inducer, doxycycline, on mice (120, 121).

Another technique that was developed to facilitate the semi-high-throughput multistrain analysis of *Mtb* mutants in a sensitive and specific manner was developed by Blumenthal et al. (122). In this work, the authors developed the use of quantitative DNA tags (qTags) that can be used to quantify mutant abundance using quantitative real-time PCR. By combining regulated gene expression with qTags, the authors found that depleting *Mtb* qTag-labeled mutants of the genes *icl*, Rv3671c, and *prcBA* in a multistrain infection of B6 mice prevented the growth and survival of the *Mtb* mutants at stages of infection concordant with single-strain infections. Importantly, the authors also showed that the quantitative analysis of the reference strains, H37Rv and Erdman, followed an expected pattern. Finally, the authors verified the utility of quantitatively measuring mutant abundance from both bacterial outgrowth on plates, as well as directly from infected lung. This method of pooling multiple *Mtb* mutants over the course of an *in vivo* infection was employed in analyzing bacterial mutant survival/virulence in a diverse panel of multiple mouse strains by Smith et al. (83). To validate their previously discussed TnSeq findings, the authors performed infections of mini-pools of qTag-labeled *Mtb* mutants *eccB1*, *mbtA*, *pstC2*, *glpK*, and *rnaseJ*, generated using oligonucleotide-mediated recombineering (ORBIT) (123), across a subpanel of CC mice, which validated the differential conditional requirement for these genes across the CC mouse panel.

### Validation: bacterial evasion of host immunity.

Verifying the role of bacterial genes in driving the host immune and pathological response remains an important goal as it can lead to the development of bacterial- and host-directed therapies. Several studies, however, have shown a discordance between cellular responses observed *ex vivo* and *in vivo* (124, 125) highlighting the importance of validation in animal models. By analyzing the immune response and disease markers of infection, various studies have verified the active role of *Mtb* in evading the host immune system in animal models, mostly through single gene knockdown *Mtb* strains. This includes a study that characterized mycobacteria carrying genomic mutations that eliminated the export of the membrane lipids PDIM and phenolic glycolipids (PGLs) to define the role of these lipids as mycobacterial effectors that recruit bacterial-permissive macrophages (51) in both zebrafish and B6 mice. The results from an *esxH* mutant study by Portal-Celhay et al. (126) suggested that the EsxHG complex might play an important role in inhibiting antigen presentation and CD4^+^ T-cell priming in mice. An extensive analysis of a Tn mutant of Rv2224c by Rengarajan et al. (127), which had been identified in a TnSeq study of mycobacterial genes essential for growth in macrophages (44), showed a vulnerability by the mutant to the immune response generated by wild-type (WT) bacteria in a mixed infection. In a monoinfection of B6 mice with the mutant, there was a significant difference in lung pathology that was not reflective of the mild reduction in CFU, further highlighting a role for this gene in the host immunopathological response. To delineate the roles of the innate and adaptive immunity to this mutant, adaptive immune-deficient RAG^−/−^ mice were infected with WT and mutant bacteria, and the results revealed an important role of innate immunity in controlling *Mtb* mutant Rv2224c growth that was lost at later time points of infection. Through the regulated targeting of essential genes *txrB2* and *birA* in mice, Su et al. (117) identified T-cell immune signatures associated with bacterial persistence/latency and gene-specific requirements for adaptive immunity control. Other studies that utilize bacterial genetic mutants to examine the role of the immune system in response to *Mtb* are reviewed by Ankley et al. (3) and Ernst (101).

Multigene or multistrain infections provide an opportunity to leverage larger scale analyses in identifying roles for various bacterial factors in immune response and disease pathology, yet these remain largely underexplored in animal models. This is primarily driven by a paucity of multigene targeting tools in the host and the difficulty in parsing specific contributions to immune response or host pathology in a multistrain infection. However, genomic barcoding tools have been utilized to assess the role of individual bacteria in the development and dissemination of granulomas in mice (128) and in macaques (129). These studies amplify the potential to examine the host immunopathological response during *Mtb* infection in multistrain experiments.

### Validation: Drug and vaccine targets.

Genes with strong essential or conditionally essential phenotypes remain a major focus for drug targets and for the development of attenuated live vaccines. As such, validating gene targets with the potential to attenuate mycobacterial growth within the host allows for the targeted development of antibacterial therapy. To validate a TnSeq study of genes involved in antibiotic response, which was discussed above, Bellerose et al. (108) ran multistrain aerosol and intravenous infections with qTag-labeled *Mtb* mutants lacking the genes found to affect bacterial fitness in each antibiotic treatment of INH, RIF, ETH, and PZA *in vivo* and showed that most mutations altered susceptibility to only one antibiotic, which faithfully replicated the results of the TnSeq study. The authors also found that many of the genes that drive *in vivo* susceptibility had little effect in an *in vitro* system, further validating the necessity of *in vivo* analysis for drug targets. These results support the strategy of identifying genes that potentiate the activity of a drug to help in identifying novel drug combination therapies. This is elegantly showcased in a study by Kalia et al. (130) in which the authors validate a synthetic lethal relationship between the clinical trial drug Q203, which targets one of the terminal oxidases of the electron transport chain (ETC) of mycobacteria (131) and the alternative ETC oxidase Cyt-*bd* in BALB/c mice.

The most direct utility of the mycobacterial genetic toolbox in developing *Mtb* vaccine therapeutics is in the development of a live, attenuated mycobacterial vaccine strain that can be optimized for infection protection in an animal model. An example of this is shown in the development and verification of MTBVAC, the first live-attenuated vaccine based on an *Mtb* human isolate to be entered into clinical trial by Arbues et al. (132). The study describes the generation of an attenuated *Mtb* strain missing the genes *phoP* and *fadD26*, the absence of which was shown to prevent the development of lethal disease in the highly susceptible SCID mouse strain similarly to the vaccine strain of BCG Danish. Further, the authors showed that MTBVAC was cleared better than BCG Danish from BALB/c mice after an intradermal injection but still induced superior protection to *Mtb* infection in intranasally infected B6 mice. In a follow-up study, Solans et al. (133) genetically inactivated an additional *Mtb* gene described as a virulence factor, *erp*, which led to even greater attenuation of this MTBVAC *erp^−/−^* strain in SCID mice compared to both MTBVAC and BCG Danish. The authors postulated the utility of this strain in high-risk individuals for whom BCG vaccination is not recommended. Several other studies have described the development of auxotrophic strains as potential vaccine candidates with protective potential in mice (134–136). As the Geneva consensus (137) stipulates requirements for a live attenuated vaccine to contain two nonreverting unmarked independent mutations, bacterial genetic tools developed to ease the generation and analysis of multiple genes in mycobacteria could significantly accelerate the development of *Mtb* vaccines.

## LOOKING FORWARD

Despite the proven utility of the bacterial genetic toolbox, there remain both a large potential and an impediment for their use in the murine model of *Mtb* infection. The continual development of bacterial tools will play a major role in identifying and validating essential *Mtb* genes *in vivo* to further our understanding of their contributions to host immunopathology and as potential antibacterial targets. Despite the largely unexplored potential of the current tools that expand the ability to target essential genes and multiple genes within a single bacterium and perform multistrain studies *in vivo*, their proven utility in *in vitro* and *ex vivo* systems suggests a tantalizing potential to speed up and ease mycobacterial gene identification and gene validation *in vivo*. Indeed, the combination of these various platforms with current genetic, proteomic, and systems biology techniques may help uncover the “black box” of *Mtb* genetics, especially as regulated within the host and considering the diversity and complexity of host response.
